# Chlorophyll reconstitution of photosynthetic light-harvesting complexes

**DOI:** 10.1093/pcp/pcaf084

**Published:** 2025-07-28

**Authors:** Yoshitaka Saga, Shota Kawato, Jiro Harada

**Affiliations:** Faculty of Science and Engineering, Department of Chemistry, Kindai University, Higashi-Osaka, Osaka 577-8502, Japan; Faculty of Science and Engineering, Department of Chemistry, Kindai University, Higashi-Osaka, Osaka 577-8502, Japan; Department of Medical Biochemistry, Kurume University School of Medicine, Fukuoka 830-0011, Japan

**Keywords:** bacteriochlorophyll, chlorophyll, light-harvesting protein, photosynthesis, pigment exchange

## Abstract

Light-harvesting complexes (LHCs) play crucial roles in efficient photoenergy conversion and photoprotection of photosynthetic systems. In LHCs, functional pigments such as chlorophylls (Chls), bacteriochlorophylls (BChls), and carotenoids are sophisticatedly assembled with the help of polypeptides. The pigment assemblies in LHCs control the site-energy of each pigment, excitonic interactions among pigments, and excitation energy gradient in the protein matrix, as well as the formation and stability of the protein structure. In vitro reconstitution of LHCs is promising in understanding these structural and functional mechanisms of LHCs. In this review, we summarize two strategies of pigment reconstitution of LHCs; one is the formation of LHCs from a mixture of photosynthetic pigments and denatured polypeptides by their self-assembly, and the other is pigment substitution by the insertion of exogenous pigments into apoproteins partially lacking bound pigments. Next, we overview reconstitution studies of major LHC II derived from oxygenic photosynthetic organisms and core and peripheral antenna proteins of purple photosynthetic bacteria. Here, we focus on substituting Chls and BChls, key pigments in photosynthesis, in LHCs by the reconstitution. (B)Chl reconstitution of LHCs has allowed us to change essential parameters for the pigment-protein interactions and photofunctions, deepening our understanding of the molecular basis of the efficient light-harvesting functions. Reconstitution of LHCs will also be helpful for the modification and design of pigment-protein complexes toward utilization of sunlight energy for global problems on agricultural productivity and bioenergy production.

## Introduction

In photosynthesis, pigment-polypeptide complexes play important roles in the efficient conversion of photoenergy to chemical energy. Photosynthetic pigment-protein complexes are divided into two groups, reaction center complexes (RCs) and light-harvesting complexes (LHCs). RCs are vital in photosynthetic systems owing to photoinduced charge separation, which is the key reaction for the accumulation of chemical potentials by multielectron redox processes (Govindjee et al. 2017). However, RCs can barely work most of the time without LHCs under usual sunlight conditions because of the dilute photon-flux density of sunlight. Harvesting of sunlight energy by LHCs in the primary stage of photosynthetic reactions is thus crucial for the efficiency of photochemical conversion in photosynthetic organisms (Croce and Amerongen 2014). LHCs have a remarkable variability in compositions of photosynthetic pigments and three-dimensional protein structures (Croce and Amerongen 2014, Saer and Blankenship 2017). Such variations of LHCs are in sharp contrast to the classification of two types of RC complexes, namely, photosystem I (PS I)-type and photosystem II (PS II)-type RCs. The diversity of LHCs originates from adaptation to different natural environments where host photosynthetic organisms grow. Most LHCs are integrated in the photosynthetic membrane although there are water-soluble LHCs that are associated with the membrane (Croce and Amerongen 2014).

In LHCs, photosynthetic pigments are densely embedded in the protein matrix except for extramembranous LHCs in green photosynthetic bacteria called chlorosomes, in which pigments are self-assembled without help of proteins (Olson 1998). The site-energy of each pigment, excitonic interaction among pigments, and pigment geometry are controlled by LHC polypeptides to optimize the light-harvesting abilities while avoiding concentration quenching (Croce and Amerongen 2014, Mirkovic et al. 2017). To unravel the mechanisms underlying the functional controls of LHCs based on the accurate combination of pigments and polypeptides, a wide variety of naturally occurring LHCs have been studied by structural and spectroscopic analysis. While the studies of native LHCs have provided fruitful information on the molecular mechanisms of photosynthetic light-harvesting, modification of LHCs by in vitro reconstitution is promising to unravel their structural and functional features from new aspects (Mimuro and Tanaka 2004, Paulsen 2006, Belgio and Ruban 2017). This strategy allows us to manipulate important parameters for the photofunctions of LHCs by substitution of pigments and amino acid residues. Such systematic modulations are difficult by investigations of native LHCs alone. In addition, reconstitution of LHCs provides detailed information on pigment-binding and folding processes of LHCs. In this review, we briefly summarize methodologies of in vitro pigment reconstitution of transmembranous LHCs and overview reconstitution studies of major LHCs by these methodologies.

## Methodologies of Pigment Reconstitution of Light-Harvesting Complexes

**Figure 1:**
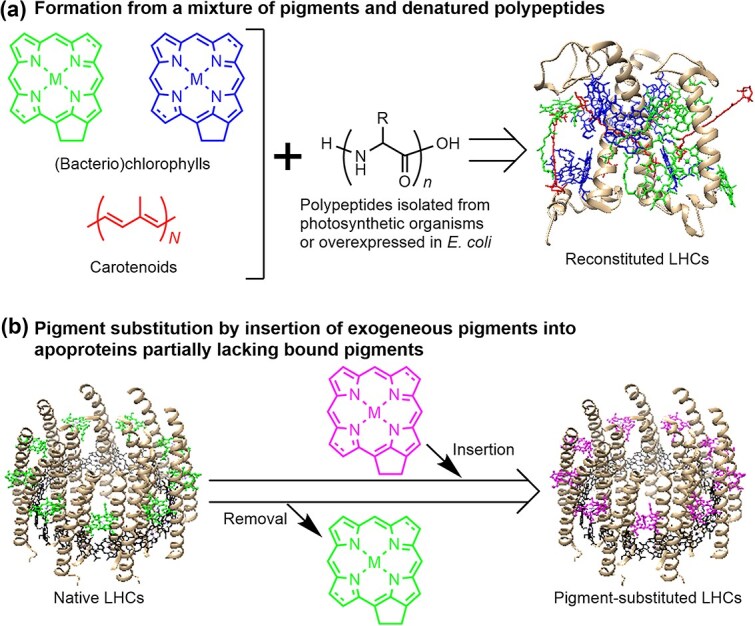
Schematic illustration of two methodologies (a and b) of pigment reconstitution of LHCs.

*In vitro* reconstitution of LHCs is a powerful strategy for the systematic manipulation of important parameters for structural and functional features of LHCs. Reconstitution of LHCs is defined here as an assembly of photosynthetic pigments with isolated polypeptides or apoproteins partially lacking pigments. Note that the term reconstitution in this review does not include the integration of LHCs into lipid bilayers, although such a description has been often used in the literature. Reconstitution methodologies of LHCs are classified into two types (Fig.  1). One is self-assembly of LHCs from a mixture of photosynthetic pigments with naturally occurring or modified polypeptides in buffer solutions (method a in Fig.  1). The typical procedure of the method a is as follows: denatured polypeptides are incubated with purified pigments in a buffer under proper renaturing conditions, followed by purification of the assembled complexes using chromatographic techniques and/or density-gradient ultracentrifugation (Croce et al. 1999a, Natali et al. 2014). This method enables changing the components (pigments and polypeptides) and their stoichiometry by control of the composition of the components at the initial step of the reconstitution experiments. Unnatural components such as synthetic pigments and polypeptides can be used to construct LHC variants and LHC-mimicking artificial complexes. Reconstitution is also advantageous to prepare minor LHCs, which have some difficulties to isolate in substantial amounts from host organisms, by using overexpressed polypeptides (Pagano et al. 1998, Ros et al. 1998, Natali and Croce 2015). The video component of the paper by Natali et al. (2014) will help in understanding the reconstitution procedure of method a.

The other reconstitution methodology is pigment substitution of LHCs by insertion of exogenous pigments into apoproteins partially lacking bound pigments (method b in Fig.  1). This method is advantageous for systematic analysis of target photosynthetic pigments in LHCs by selective pigment exchange in the protein with no change in the other components and geometries. Reconstitution by method b is performed by a combination of partial removal of bound pigments from native LHCs and subsequent insertion of exogeneous pigments into the empty pigment-binding pockets. Method has been applied to LH2 proteins derived from purple photosynthetic bacteria (Paulsen 2006). Selective pigment removal from native LH2 is a key step in the reconstitution by method b, and protocols for this have been established for LH2 from some species of purple bacteria (Bandilla et al. 1998, Fraser et al. 1999, Saga et al. 2017, 2018a, Kawato et al. 2024).

Polypeptides for reconstitution in method a are prepared by isolation from native and mutated LHCs or heterogeneous overexpression in *E. coli*. Native and mutated polypeptides are generally obtained from purified LHCs or photosynthetic membranes by pigment removal with organic solvents. In a while, heterogeneous overexpression of LHC polypeptides is performed using an overexpression vector containing the target polypeptide gene, which is transformed into *E. coli*, followed by collection of the LHC polypeptide after disruption of the *E. coli* cells. The overexpressed polypeptide of transmembranous LHCs is typically present in inclusion bodies. These LHC polypeptides are solubilized from the inclusion bodies with detergents in reconstitution experiments. Overexpression of LHC polypeptides is advantageous for fusion with a histidine-tag (His-tag) for easy purification of reconstituted LHCs. In addition, site-directed mutation of LHC polypeptides can be achieved by heterogeneous overexpression. Solid-phase peptide synthesis has also been used for the preparation of polypeptides for the reconstitution of LHCs.

**Figure 2:**
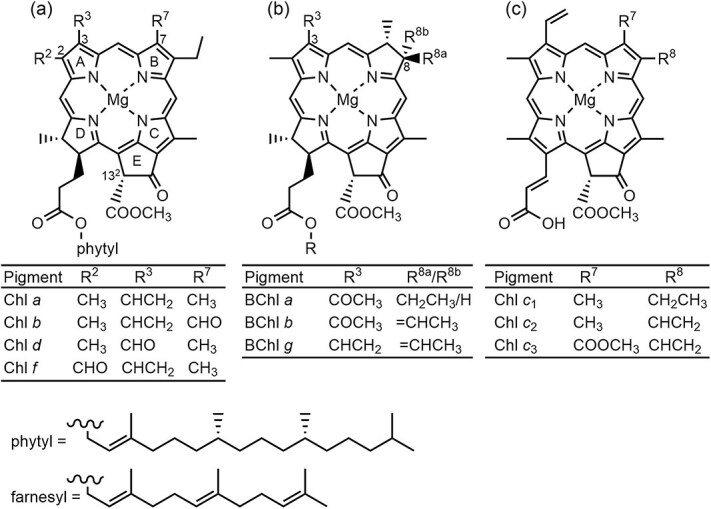
Molecular structures of naturally occurring Chls and BChls. (a) Chlorin-type pigments in oxygenic photosynthetic organisms. (b) Bacteriochlorin-type pigments in photosynthetic bacteria. (c) Porphyrin-type pigments in chromophyte algae and some prokaryotes. Molecular structures of chlorosomal BChls in green photosynthetic bacteria are shown in Fig. S1.

Three types of highly π-conjugated molecules, namely cyclic tetrapyrrole, linear polyene (carotenoid), and open tetrapyrroles (phycobilin), are engaged in LHCs as light-absorbers. Among them, cyclic tetrapyrrole pigments, namely chlorophyll (Chl) and bacteriochlorophyll (BChl), play central roles in the photofunctions of LHCs (Chen 2014, Simkin et al. 2022, Tamiaki and Kichishima 2025). Natural Chl and BChl pigments show a structural diversity in tetrapyrrole skeletons and peripheral substituents (Fig.  2). The cyclic tetrapyrrole structure of Chl pigments, except Chls *c*, is a chlorin macrocycle (17,18-dihydroporphyrin). Chls *c* in chromophyte algae and some prokaryotes have a fully π-conjugated porphyrin skeleton. The photofunctional core of BChl pigments (BChls *a*, *b*, and *g*) in bacterial photosynthesis, such as purple bacteria and heliobacteria, consists of a bacteriochlorin skeleton (7,8,17,18-tetrahydroporphyrin). Conversions of the cyclic tetrapyrrole structures from porphyrin to chlorin and from chlorin to bacteriochlorin largely shift a lowest-energy absorption band called Q_y_ band and increase its molar extinction coefficients. Peripheral substituents on the cyclic tetrapyrroles of Chl and BChl pigments also show a remarkable diversity and contribute to the tuning of their spectral features. The wide variation of the spectral features of (B)Chls, while little change in the molecular size, is advantageous to control the physicochemical features of LHCs by the reconstitution strategy. Therefore, reconstitution of (B)Chls bound to LHCs has been applied to investigate the photofunctional mechanisms of LHCs. This review focuses on (B)Chl reconstitution of major LHC II of oxygenic photosynthetic organisms and LH1-LH3 of purple photosynthetic bacteria.

## Light-Harvesting Complexes of Oxygenic Photosynthetic Organisms

### LHC II

LHC II (major LHC II) is an abundant light-harvesting pigment-polypeptide complex associating with PS II in land plants and green algae. The polypeptide of LHC II forms three transmembrane α-helices with two short helices on the lumen side and assembles eight Chl *a*, six Chl *b*, and four carotenoids (Liu et al. 2004, Standfuss et al. 2005). Chl *b* and carotenoids transfer excitation energy to Chl *a* in LHC II. Carotenoids also play important roles in the dissipation of excess excitation energy. LHC II is a promising photosynthetic protein to study molecular mechanisms underlying structural formation by assembly of pigments with polypeptides and intracomplex excitation energy flow among different kinds of pigments in the protein matrix. In this regard, in vitro reconstitution of LHC II has been studied by method a in Fig.  1. At the early stage of the reconstitution studies, LHC II polypeptides isolated from thylakoid membranes were used (Plumley and Schmidt 1987). However, reconstitution from native polypeptides has difficulty in the separation of LHC II proteins consisting of different amino acid sequences and a potential necessity to separate from coexisting minor LHCs. Reconstitution of LHC II using overexpressed polypeptides overcomes these problems. Paulsen and coworkers developed reconstitution of LHC II from polypeptides overexpressed in *E. coli* (Paulsen et al. 1990, Paulsen and Hobe 1992).

The reconstitution of LHC II provides valuable insights into the binding features and structural roles of Chls and carotenoids in this protein. Earlier studies indicated the importance of carotenoids, which are necessary for the structural formation of LHC II (Plumley and Schmidt 1987, Paulsen et al. 1990, Paulsen and Hobe 1992, Croce et al. 1999a). Reconstitution studies indicated the high affinities of lutein and neoxanthin in the L1/L2 and N1 sites, respectively, in LHC II (Croce et al. 1999a, 1999b, Hobe et al. 2000). Chls are also crucial for the structural formation of LHC II. Especially, Chl *b* is required for stabilization of the LHC II structure (Horn and Paulsen 2004). The stoichiometry of Chls *a* and *b*, their binding affinities, and assembling process were thoroughly investigated (Kleima et al. 1999, Remelli et al. 1999, Horn and Paulsen 2002, 2004, Hobe et al. 2003, Horn et al. 2007). Among six binding sites of Chl *b* in LHC II, five binding sites were revealed to be specific to Chl *b*, and one site exhibits a slight preference for Chl *b* rather than Chl *a* (Hobe et al. 2003). The folding of LHC II in vitro occurred with the coupling of pigment assembly and secondary structure formation of the polypeptide in two kinetic phases (Horn and Paulsen 2002, 2004, Horn et al. 2007, Dockter et al. 2009). The initial phase of the folding of LHC II is the preferential binding of Chl *a* with the polypeptide. Next, Chl *b* slowly occupies Chl-binding sites, including the specific Chl *b* sites. The promiscuous Chl binding sites are suggested to be occupied by both Chls *a* and *b* reversibly until stabilization of the pigment-polypeptide complex. The binding of Chl *b* in the late phase stabilizes the LHC II structure from the labile complex. Reconstitution using mutated polypeptides has also provided insights into the roles of several amino acid residues in Chl binding and stability of LHC II (Heinemann and Paulsen 1999, Yang et al. 1999, Mick et al. 2004).

**Figure 3:**
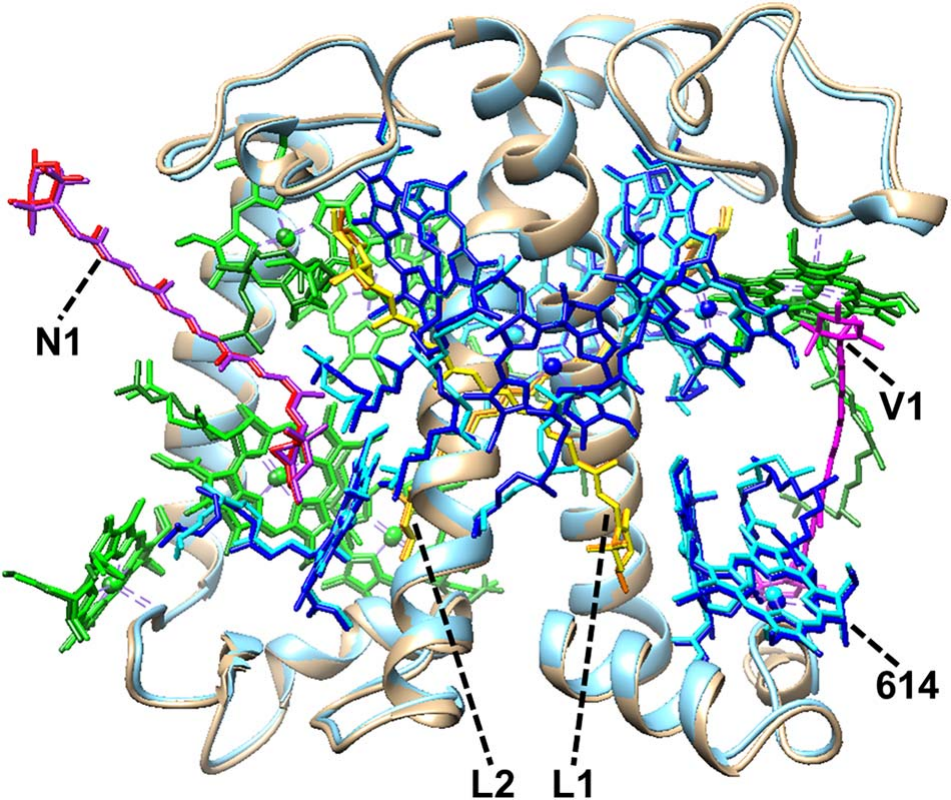
Structural comparison between native LHC II (PDB entry, 8Y15) and reconstituted LHC II (PDB entry, 8YEE). Chl *a*, Chl *b*, lutein, neoxanthin, and carotenoid at the V1 site in native LHC II are colored blue, forest green, yellow, red, and magenta, respectively. Chl *a*, Chl *b*, lutein, and neoxanthin in reconstituted LHC II are colored cyan, green, orange, and purple, respectively. Polypeptides in native LHC II and reconstituted LHC II are colored gold and sky blue, respectively. Chl *a* is assigned as a representative at site 614 in the structure of reconstituted LHC II.

Successful reconstitution of LHC II has been typically judged from similarities of biochemical, spectroscopic, and functional features between reconstituted LHC II and native LHC II. In such situations, the three-dimensional structure of reconstituted LHC II was resolved by cryo-electron microscopy (cryo-EM) (Seki et al. 2024). The overall protein structure of reconstituted LHC II was essentially the same as native LHC II (Fig.  3), but some amino acid residues in the C-terminal region were not structurally determined, probably due to aggregation of the His-tags. Such perturbation by the His-tags suggests a necessity to pay attention to the use of reconstituted LHC II in the studies on nonphotochemical quenching (NPQ) concerning the C-terminal region (Liguori et al. 2016, Kondo et al. 2017). The binding of Chls in reconstituted LHC II almost matched well with that in native LHC II (Table 1). The mixed occupancy of Chl *a* and Chl *b* in site 614 in reconstituted LHC II is in line with the minor selectivity of this site indicated by previous reconstitution (Remelli et al. 1999) and molecular dynamic simulation studies (Elias et al. 2023). A large difference in the pigment binding is the lack of carotenoid in the V1 site in reconstituted LHC II. This might be ascribed to the low affinity of carotenoid in the V1 site in LHC II. This study gives validity to the in vitro reconstitution strategy for investigations of LHC II from the structural aspect.

**Table 1:** Summary of Chl occupancy in native LHC II and reconstituted LHC II by Cryo-EM analysis (Seki et al. 2024), native LHC II by X-ray crystallographic analysis (Liu et al. 2004), and predicted by MD simulation (Elias et al. 2023)

Site	Native LHC II^a^	Reconstituted LHC II^a^	Native LHC II^b^	Prediction^c^
601	Chl *b*	Chl *b*	Chl *b*	Chl *b*
602	Chl *a*	Chl *a*	Chl *a*	Chl *b*
603	Chl *a*	Chl *a*	Chl *a*	mixed
604	Chl *a*	Chl *a*	Chl *a*	mixed
605	Chl *b*	Chl *b*	Chl *b*	Chl *a*
606	Chl *b*	Chl *b*	Chl *b*	Chl *b*
607	Chl *b*	Chl *b*	Chl *b*	Chl *b*
608	Chl *b*	Chl *b*	Chl *b*	Chl *b*
609	Chl *b*	Chl *b*	Chl *b*	Chl *b*
610	Chl *a*	Chl *a*	Chl *a*	Chl *a*
611	Chl *a*	Chl *a*	Chl *a*	mixed
612	Chl *a*	Chl *a*	Chl *a*	Chl *b*/mixed
613	Chl *a*	Chl *a*	Chl *a*	Chl *b*
614	Chl *a*	mixed	Chl *a*	Chl *b*

a Seki et al. 2014

b Liu et al. 2004

c Elias et al. 2023.

The target of most reconstitution studies of LHC II has been monomeric LHC II although LHC II trimers act as photon absorbers in the thylakoid membrane. The reconstituted LHC II monomers have significantly contributed to deepening our understanding of the structural and functional features of LHC II, as described above. In contrast, *in vitro* formation of LHC II trimers has been scarcely studied. Reconstitution of LHC II trimers should be developed to unravel the mechanisms of monomer assembly and *in vivo* photofunctions. The trimer formation is also important for structural analysis by Cryo-EM (Seki et al. 2024).

Reconstitution of LHC II has been utilized to elucidate its EET and NPQ dynamics by a combination of time-resolved spectroscopy (Croce et al. 2001, Polívka et al. 2002, Natali et al. 2016). In addition to the reconstitution for fundamental studies on the photofunctions of LHC II, reconstitution toward artificial systems for photoenergy utilization has been progressed. One trend is the modification of LHCs and the expansion of their absorption range using the reconstitution methodology. Substitution of Chl *a* to Chl *d* and Chl *f*, both of which exhibit red-shifted Q_y_ absorption bands, in LHC II and LHC I was successfully performed by in vitro reconstitution (Elias et al. 2021, 2024, Cianfarani et al. 2025). Chl *d* and Chl *f* were bound instead of Chl *a* in these LHCs and newly exhibited far-red absorption bands while maintaining the native architecture. The reconstitution of Chl *d* and Chl *f* did not interfere photofunctions of the LHCs. The utilization of far-red light by such pigment substitution in LHCs potentially increases the photosynthetic activity of oxygenic photosynthesis driven by visible light. Attachment of organic dyes with reconstituted LHC II is an alternative strategy increasing the absorption cross section (Wolf-Klein et al. 2002, Gundlach et al. 2009, Peneva et al. 2010). Reconstitution using LHC II polypeptides, in which a cysteine residue was introduced, and conjugation of dyes via this residue successfully yielded biohybrid LHC II possessing artificial dyes, which served as energy donors or acceptors against Chls in the protein. Attachments of organic dyes absorbing green light, which is less captured by native LHC II, have allowed this protein to utilize green light efficiently. Another trend is a combination of reconstituted LHC II with inorganic materials. Reconstituted LHC II possessing oligopeptide tags was able to bind quantum dots, and excitation energy transfer occurred between Chls in the protein and quantum dots (Werwie et al. 2014). Reconstituted LHC II was also immobilized on a gold surface with self-assembled monolayers via His-tag and characterized by surface-plasmon resonance field fluorescence spectroscopy (Liu et al. 2008). Furthermore, reconstituted LHC II was applied to dye-sensitized solar cells for photoelectrochemical conversion (Lämmermann et al. 2019). The combination of LHC II with inorganic materials occasionally enhanced the stability of LHC II (Roeder et al. 2014, Werwie et al. 2014). These results will be a clue to the expansion of the photofunctional ability of LHC II and the development of artificial photoenergy conversion systems using LHC II.

## Light-harvesting complexes of purple photosynthetic bacteria

### LH1

Purple photosynthetic bacteria have LH1 as a core antenna protein. LH1 encircles RC and forms a stoichiometric LH1-RC complex. LH1 accepts excitation energy from peripheral antenna LH2 and transfers the excitation energy to RC in the photosynthetic membrane of purple bacteria. LH1 consists of BChls, carotenoids, and transmembrane polypeptides. Basically, in a subunit of LH1 complex, two BChls, which form a dimer and are tightly coupled with each other, and one or two carotenoids are organized with transmembrane α- and β-polypeptides. Note that the stoichiometry of the components of LH1 subunits has a diversity arising from additional BChls and polypeptides (Qian et al. 2018, Xin et al. 2018, Wang et al. 2024, Tani et al. 2025). The LH1 subunits are circularly arranged to form a closed or an open ring (Qian et al. 2018, Xin et al. 2018, Yu et al. 2018, Tani et al. 2020, 2022, 2024, Bracun et al. 2021). The numbers of the subunits in an LH1 complex are generally distributed from 14 to 17 (Swainsbury et al. 2023), although the subunit numbers are fewer than 14 in certain species (Tani et al. 2023). LH1 exhibits a Q_y_ absorption band in the near-infrared region ranging from 860 nm to 1020 nm, depending on the species of purple bacteria (Kimura et al. 2023). The spectral diversity and EET dynamics of LH1 based on the pigment components and the protein structure are clues to the utilization of near-infrared light. Reconstitution of LH1 gains insights into its structural and functional features that allow purple bacteria to resource near-infrared light energy. It is worth noting that the reconstitution of LH1 has contributed to obtaining the structural information since the LH1 structure at the atomic level was resolved much later than those of other major LHCs such as LHC II and LH2 (Niwa et al. 2014).

Reconstitution of LH1 by method a in Fig.  1 is performed by mixing pigments and transmembrane polypeptides in the presence of a detergent (*n*-octyl-β-D-glucoside is usually used) in a buffer, followed by dilution and cooling of the mixed solution. BChl *a* and polypeptides are assembled stepwise in this reconstitution process; the subunit-type species exhibiting a Q_y_ band at around 820 nm (B820) is formed, and further assembly occurs to form LH1-type species with around 870 nm absorption (Parkes-Loach et al. 1988). CD spectroscopy of reconstituted LH1 confirms the formation of the complex that resembles native LH1. The β-polypeptide alone with BChl *a* formed the B820 species, although the α-polypeptide was necessary for the LH1-type complex (Parkes-Loach et al. 1988). The α-polypeptide alone with BChl *a* cannot form both the B820 and LH1-type species. Polypeptides for reconstitution of LH1 were prepared by isolation from native (Parkes-Loach et al. 1988, Loach et al. 1994, Davis et al. 1995, Parkes-Loach et al. 2004) or mutagenized proteins (Davis et al. 1997, Todd et al. 1999), heterogeneous overexpression in *E. coli* (Dewa et al. 2005), and solid-phase peptide synthesis (Kehoe et al. 1998, Meadows et al. 1998, Parkes-Loach et al. 2004). The various methods for preparations of LH1 polypeptides, including peptide cleavages (Meadows et al. 1995), have enabled to analyze the required amino acid sequences for the reconstitution. Such analysis was extensively performed for the β-polypeptide of LH1 from *Rhodobacter* (*Rba*.) *sphaeroides* and *Rhodospirillum* (*Rsp*.) *rubrum*. Fig. S2 summarizes examples of amino acid sequences of chemically synthesized polypeptides as analogs of the LH1 β-polypeptide of *Rba. sphaeroides*. A short polypeptide consisting of the last 16 amino acids of the β-polypeptide (*sphaeroides* β16) did not form either a subunit-type or LH1-type complex (Meadows et al. 1998). In contrast, elongated polypeptides consisting of the last 31 and 37 amino acids (*sphaeroides* β31 and *sphaeroides* β37, respectively) formed a subunit-type complex showing a Q_y_ band at around 820 nm with themselves or with native α-polypeptide (Kehoe et al. 1998, Meadows et al. 1998). Further elongation (*sphaeroides* β41) formed an LH1-type complex (Kehoe et al. 1998). A similar tendency was observed in the analysis of LH1 polypeptide analogs of *Rsp. rubrum*. Substitution of conserved amino acid residues of the LH1 β-polypeptide also provided information on their roles in assemblies and spectral features of both subunit-type and LH1-type complexes (Davis et al. 1997, Kehoe et al. 1998, Todd et al. 1999, Parkes-Loach et al. 2004).

Structural requirements of cyclic tetrapyrrole pigments for binding to the LH1 polypeptide were indicated by reconstitution assays using various (B)Chl derivatives (Parkes-Loach et al. 1990). Central metal and acetyl and methoxycarbonyl groups at the 3- and 13^2^-positions, respectively, in BChl *a* (see Fig.  2) were required for the reconstitution. Replacement of the central magnesium in BChl *a* to nickel did not prevent the reconstitution (Fiedor et al. 2000, 2001). Ni-BChl *a* in reconstituted LH1 can serve as a probe to analyze the excitonic state of coupled BChl *a* ring array owing to ultrafast deactivation of the excited state of Ni-BChl *a*. Information on the structural requirements of polypeptides and pigments for LH1 assembly has been utilized for developments of LH1-mimicking pigment-polypeptide complexes (Nango et al. 2002, Noy and Dutton 2006, Ochiai et al. 2008, Springer et al. 2012, Reddy et al. 2013).

Although the reconstitution of LH1 has generally been analyzed using near-infrared absorption spectroscopy, structural analysis has provided insights into the subunit-type B820 complex. Solution and solid-state nuclear magnetic resonance (NMR) spectroscopy can detect NMR signals of dimeric ^13^C-labelled BChl *a* in the subunit and obtain information on dimetric conformation of BChl *a* molecules with a partial overlap of the macrocycle and hydrogen-bonding of BChl *a* with the polypeptides (Wang et al. 2002, Umetsu et al. 2005). Small-angle neutron scattering (SANS) analysis revealed that the reconstituted subunit-type complex indeed consisted of two BChl *a* molecules with a pair of α- and β-polypeptides and BChl *a* was crucial for stabilizing the complex (Wang et al. 2003). The SANS analysis also indicated a difference in the assembling tendency between α- and β-polypeptides, probably correlating with different behaviors between the two polypeptides in the reconstitution of LH1.

### LH2 and LH3

**Figure 4:**
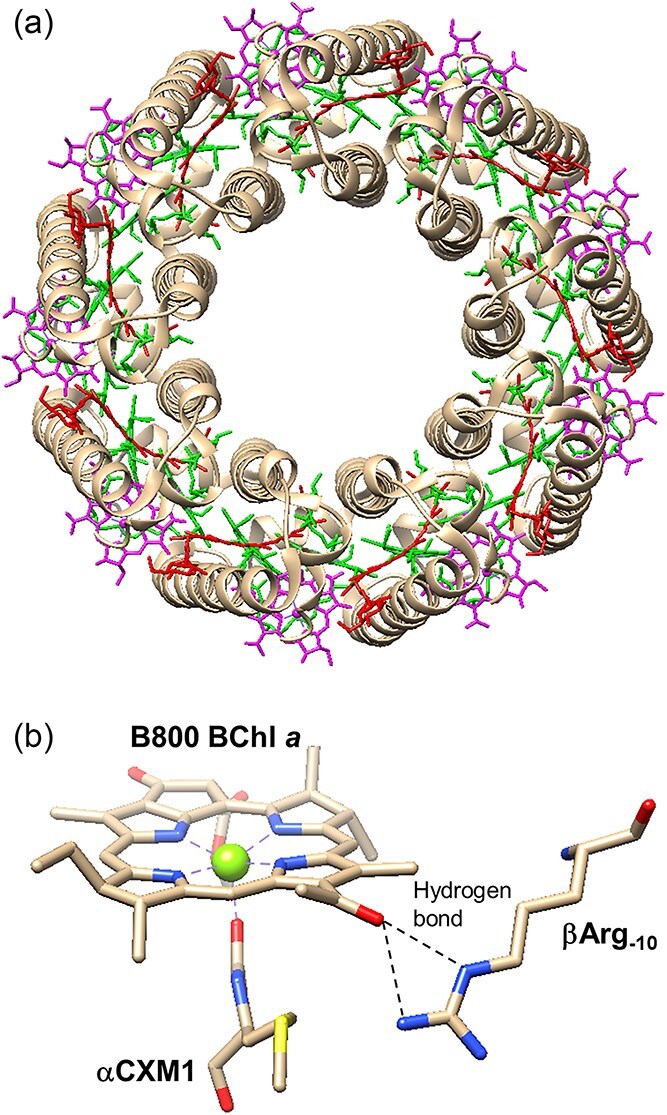
Top-view overall structure (a) and major interactions of B800 BChl *a* with neighboring amino acid residues (b) in *acidophilus*-LH2 (PDB entry, 1NKZ).

LH2 is a peripheral light-harvesting protein in purple photosynthetic bacteria. This protein is formed by the circular arrangement of repeating pigment-polypeptide subunits in the photosynthetic membrane (Fig.  4a). The ring sizes of LH2 by the assembly of repeating subunits depend on host bacteria (McDermott et al. 1995, Koepke et al. 1996, Gardiner et al. 2021, Qian et al. 2021, Morimoto et al. 2023). Each subunit contains BChl *a* and carotenoid with a pair of transmembrane α- and β-polypeptides. BChl *a* pigments in LH2 proteins are classified into two types, termed B800 and B850 BChl *a*, referred to the positions of the Q_y_ absorption band (Cogdell et al. 2006). B800 BChl *a* has a monomeric character and shows its Q_y_ band at around 800 nm. In contrast, B850 BChl *a* pigments are present as a dimeric form in a subunit and interact excitonically in a subunit as well as in the circular arrangement. As a result of the excitonic interactions, the Q_y_ band of B850 BChl *a* is positioned at around 850 nm. Rapid and efficient EET from B800 to B850 BChl *a* in LH2 is one of the representative systems to study photosynthetic energy flow mechanisms (Sundström et al. 1999, Sholes et al. 2011, Mirkovic et al. 2017).

Reconstitution by method b in Fig.  1 has been applied to the substitution of B800 BChl *a* in LH2. In the first step of the B800 reconstitution, B800 BChl *a* is selectively removed from native LH2 solubilized with *n*-dodecyl-β-D-maltoside under acidic pH conditions in the presence of additional detergent (Bandilla et al. 1998, Fraser et al. 1999, Saga et al. 2017, 2018a, Kawato et al. 2024). The prepared LH2 lacking B800 BChl *a* (B800-depleted LH2) keeps the overall ring structure and the local structures such as the molecular orientation of B850 BChl *a* and the secondary structure of transmembrane polypeptides (Saga et al. 2017). In the second step, exogeneous (B)Chl pigments are inserted into the B800-binding pockets by incubation of B800-depleted LH2 with the pigments under neutral pH conditions. Proper accommodation of exogenous BChl *a* in the B800-binding pocket was confirmed by means of electronic absorption, circular dichroism, and resonance Raman spectroscopy (Gall et al. 1999, Saga et al. 2017), indicating a validity of the B800 reconstitution for the studies of LH2. It is noted that the spectroscopic analysis provided useful information on the excitonic interactions, molecular orientations, and hydrogen-bonding patterns of exogeneous BChl *a* in the B800 pocket, but detailed structural analysis will be required to study the pigment state and functions in the B800-binding pocket precisely. It was reported that the B800-binding pocket of LH2 from *Rhodoblastus* (*Rbl.*) *acidophilus* and *Rba. sphaeroides* (denoted as *acidophilus*-LH2 and *sphaeroides*-LH2, respectively) can accept various bacteriochlorin-type (BChl *a*, BChl *b*, 3-vinyl BChl *a*, 3^1^-OH BChl *a*, and Zn BChl *a*, see Fig.  2 and Fig. S3), chlorin-type (Chl *a*, Chl *b*, Chl *d*, Chl *f*, 3-acetyl Chl *a*, and pyroChl *a*), and porphyrin-type pigments (3-acetyl protoChl *a*) (Bandilla et al. 1998, Fraser et al. 1999, Saga and Miyagi 2018, Saga et al. 2018b, 2018c, 2019, 2020, 2021, 2022, Swainsbury et al. 2019). These results suggest that alterations in the substituents in the A- and B-rings of the cyclic tetrapyrrole and the dehydrogenation of the B-ring (bacteriochlorin → chlorin) are allowable in the B800-binding pocket of LH2. Note that successful reconstitution by method a in Fig.  1 is limited to LH2 from *Phaeospirillum* (*Phs*.) *molischianum* (Todd et al. 1998), and thus there is no information on in vitro substitution of B850 BChl *a*.

Substitution of B800 BChl *a* in LH2 can systematically modulate the spectral feature of an energy donor and intracomplex EET in LH2. Table 2 summarizes the Q_y_ peak positions and downshifts of the Raman signals of the carbonyl group on the A-ring (3-C=O or 2-C=O, see Fig.  2) of (B)Chl pigments in the B800-binding site. The two spectral features of the substituted pigments in the B800 site are focused here since the 3-C=O Raman signals of BChl *a*, which reflect hydrogen-bonding of BChl *a* with neighboring amino acid residues, are correlated with the Q_y_ positions of BChl *a* in LH proteins from purple photosynthetic bacteria (Kimura et al. 2023). The tendency of the Q_y_ red-shift of (B)Chls by insertion into the B800-binding pocket is classified into three types. The first type involves (B)Chl pigments possessing the C=O group at the 3-position of the cyclic tetrapyrrole, namely BChl *a*, BChl *b*, and Chl *d*. These pigments exhibit a large Q_y_ shift (430–560 cm^−1^) by insertion into the B800-binding pocket. The 3-C=O stretching vibrational bands of the three (B)Chls are downshifted from the monomeric form. The downshifts of the 3-C=O bands indicate that the 3-C=O moiety of these (B)Chls is hydrogen-bonded with the conserved arginine residue at position −10 (βArg_−10_) with respect to the B850 ligand histidine of the LH2 β-polypeptide (Supplementary Fig. S4). This hydrogen-bonding is essentially the same as that of the 3-acetyl group in B800 BChl *a* with βArg_−10_ in native LH2 (Fig.  4b). Therefore, the Q_y_ red-shift of these (B)Chls in the B800 site is ascribable to the hydrogen-bonding of the 3-C=O group in a similar manner to native LH2. The second type of pigments are Chl *a* and Chl *b*, which have no C=O group at the 3-position. Both the Chls show small red-shifts of the Q_y_ band (96–180 cm^−1^) because of no hydrogen-bonding. The third type is Chl *f* that possesses the 2-C=O group, exhibiting moderate Q_y_ red-shift and a large downshift of the 2-C=O stretching vibrational band. These results indicate that the Q_y_ red-shift of Chl *f* by insertion into the B800-binding pocket is majorly due to the hydrogen-bonding of the 2-C=O group. However, the Q_y_ red-shift of Chl *f* was smaller than that of (B)Chls classified in the first type. The difference might arise from the different hydrogen-bonding patterns and/or the steric hindrance of the 3-vinyl group in Chl *f*.

**Table 2:** Summary of Q_y_ peak positions of (B)Chl pigments in the B800-binding pocket of *acidophilus*-LH2 and downshifts of the 2- or 3-C=O Raman signals of (B)Chls by insertion into the B800-binding pocket

(B)Chl	λ(Q_y_) in the B800 site/nm	Q_y_ red-shift ^a^/cm^−1^	Downshift ^b^/cm^−1^	Reference
BChl *a*	802	518	35	Saga et al. 2022
BChl *b*	831	561	37	Saga et al. 2021
Chl *a*	670	180	– ^c^	Saga et al. 2018b
Chl *b*	649	96	– ^c^	Saga et al. 2019
Chl *d*	710	429	39	Saga et al. 2018b
Chl *f*	711	283	42	Saga et al. 2022

a Q_y_ red-shift = 1/λ (in acetone)—1/λ (in the B800 cavity).

b Downshift = ν (monomer)—ν (in the B800 cavity).

c Absence of the 2- or 3-C=O group.

Reconstitution of (B)Chls into the B800 binding pocket can investigate the effect of the Q_y_ band of the energy donor on intracomplex EET in LH2 without change in the structural relationship between the energy donor and acceptor (Herek et al. 2000, Swainsbury et al. 2019, Saga et al. 2021). Table 3 summarizes EET rate constants from (B)Chls in the B800-binding pocket to B850 BChl *a* in LH2. Intracomplex EET from B800 to B850 BChl *a* occurs in sub-picosecond timescale. A similar EET rate constant was observed in Zn BChl *a*-reconstituted LH2, which showed a slightly blue-shifted Q_y_ band of the energy donor. Substitution of B800 BChl *a* with BChl *a* analogs (3^1^OH-BChl *a* and 3-vinyl BChl *a*), whose Q_y_ bands are blue-shifted by ca. 40–50 nm, somewhat retarded intracomplex EET. In contrast, Chl-reconstituted LH2 exhibited further slow EET, and the rate constants depended on the Q_y_ positions of Chls in the B800 site.

**Table 3:** Summary of the EET rate constants from (B)Chls in the B800-binding pocket to B850 BChl *a* in reconstituted LH2

(B)Chl	Rate constant/ps	λ(Q_y_) in the B800 site/nm	Reference
BChl *a*	0.9	800	Herek et al. 2000
	0.5	800	Swainsbury et al. 2019
	0.96	802	Saga et al. 2021
Zn BChl *a*	0.8	794	Herek et al. 2000
3-vinyl BChl *a*	1.4	765	Herek et al. 2000
3^1^-OH BChl *a*	1.8	753	Herek et al. 2000
Chl *f*	2.7	ca. 710	Swainsbury et al. 2019
Chl *d*	3.5	704	Swainsbury et al. 2019
3-acetyl Chl *a*	4.4	694	Herek et al. 2000
	5.1	691	Swainsbury et al. 2019
Chl *a*	8.3	670	Herek et al. 2000
	6.7	666	Swainsbury et al. 2019
BChl *b*	≤ 0.1	829	Swainsbury et al. 2019
	0.91	831	Saga et al. 2021

Rapid EET from B800 to B850 BChl *a* in native LH2 can be explained by an EET pathway via higher-lying optically forbidden excitation states of the circular B850 aggregate (Mukai et al. 1999, Scholes et al. 2000). This mechanism reveals that overlap of the density of states (DOS) of B850 BChl *a* including the higher-lying optically forbidden excitation states with a luminescence band of energy-donating (B)Chls in the B800 site is responsible for EET in reconstituted LH2. The Q_y_ blue-shifts of the energy donor by insertion of BChl *a* analogs decrease this overlap, resulting in slower EET than native LH2. In Chl-reconstituted LH2, however, luminescence bands of Chls in the B800 site hardly overlap with DOS of B850 BChl *a*. Therefore, overlap with the long tail of the B850 band, which probably originates from the vibronic manifolds of the highest excitonic eigenstate of B850 BChl *a*, is rather responsible for EET in Chl-reconstituted LH2 (Herek et al. 2000). Such overlap is much smaller than the overlap in native LH2. Therefore, slow intracomplex EET in Chl-reconstituted LH2 is interpreted by large decreases in the spectral overlap integral between energy-donating Chls and 850 BChl *a*.

There is a discrepancy for the EET rate in BChl *b*-reconstituted LH2; its EET was reported to be similar to that in native LH2 for *acidophilus*-LH2 (Saga et al. 2021) but significantly fast (≤ 0.1 ps) for *sphaeroides*-LH2 (Swainsbury et al. 2019). The reason for this discrepancy is unclear, although the discrepancy might originate from an inherent difference between *acidophilus*-LH2 and *sphaeroides*-LH2. The EET rate of BChl *b*-reconstituted LH2 analogous to native LH2 (Saga et al. 2021) is consistent with that of LH3, which is a spectral variant of LH2 and increases a spectral overlap between the energy donor and acceptor owing to blue-shift of the Q_y_ band of the energy acceptor (B820 BChl *a*), with the EET rate constant of 0.7–0.9 ps (Kennis et al. 1997, Ma et al. 1998, Tong et al. 2020). The similarity of the EET rate of BChl *b*-reconstituted LH2 with native LH2 despite an apparent increase of the spectral overlap integral between the energy donor (BChl *b*) and the acceptor (B850 BChl *a*) can be rationalized by the contribution of the DOS of B850 BChl *a* to EET. Taking the overlap integral of the B850 DOS with luminescence of energy-donating BChls into account, the Q_y_ red-shift of the energy donor in the B800 site by insertion of BChl *b* does not notably accelerate EET in LH2.

LH3, which expresses under low-light conditions in some species of purple photosynthetic bacteria (Gardiner et al. 2018), has attracted attention because of energy-accepting excitonically coupled B820 BChl *a*, whose Q_y_ band is shifted to a shorter wavelength than that of B850 in LH2. The ring structure of LH3 is essentially the same as that of LH2 derived from the same bacteria (McLuskey et al. 2001). The different Q_y_ spectral feature of B820 from B850 is majorly explained by hydrogen-bonding patterns of the 3-acetyl group of BChl *a* with neighboring amino acid residues (Cogdell et al. 2002, Montemayor et al. 2018, Ramos et al. 2019). The substitution of B800 BChl *a* in LH3 benefits to deepening our understanding of EET mechanisms of peripheral antenna proteins of purple bacteria. From this viewpoint, the removal of B800 BChl *a* from LH3 was examined under acidic conditions. Despite the structural similarity between LH3 and LH2, B800 BChl *a* in LH3 is tolerant to the removal from the protein compared with B800 in LH2 (Saga et al. 2025). In addition, LH3 tended to decompose through the B800 removal process. These results imply that the LH3 structure is somewhat more unstable and flexible than the LH2 structure. These results will be helpful for the developments of reconstitution of LH3 by method b.

## Perspective

Pigment reconstitution of LHCs is a powerful strategy to study the molecular mechanisms underlying the assembly of photosynthetic pigments with polypeptides, control of site energies and excitonic interactions of the bound pigments, and excitation energy flow inside the protein. The reconstitution methodologies of many LHCs have been established, allowing us to elucidate the structural principles and functional mechanisms of LHCs. At present, reconstitution of peripheral LHCs (LH2 and LH3) from purple photosynthetic bacteria is difficult by method a in Fig.  1. A reason for unsuccessful reconstitution of these LHCs by method A should be unraveled.

The current reconstitution of LHCs, which is based on simple self-assembly of pigments and polypeptides under proper renaturing conditions, has some limitations for precise control of folding fidelity, pigment selectivity, and excitonic interactions. In the current reconstitution, one can control only the initial conditions such as the kinds and stoichiometry of the components (pigments and polypeptides) and detergent types and concentrations, and primitive conditions in the folding process such as incubation time and temperature. A deep understanding of the intermediate states and dynamics in the reconstitution process, such as transient interactions between pigments and polypeptides, roles of detergents in the folding process, and kinetic and thermodynamic parameters of the intermediate complexes will overcome the limitations. Control of the intermediate pigment-polypeptide complexes based on the understanding of these features will also help to increase the reconstitution efficiency.

Formation of multimers of LHCs such as LHC II trimers and supercomplexes consisting of several LHCs and RCs is also limited in the current reconstitution studies. Control of the interactions among multiple complexes will be a clue in the formation of the LHC network in photosynthetic systems. The methodology of multimer formation will also have a potential to make the diverse ring structures of LH1. Computational design of pigment-protein complexes (Ennist et al. 2024) will be helpful for the precise control of the reconstitution of LHCs at the monomer, multimer, and supercomplex levels.

Structural verification of reconstituted LHCs at the atomic level is also important, although spectroscopic and biochemical assays have typically been applied to judge successful reconstitution of LHCs. Recent progress in structural biology will allow us to determine the atomic-level structures of reconstituted LHCs. The multimer formation of the reconstituted LHCs will be a key technique for cryo-EM analysis.

From the viewpoints of functional analysis, a combination of advanced functional spectroscopic techniques such as two-dimensional spectroscopy with in vitro reconstitution strategy, which enables systematic changes of important parameters for the photofunctions of LHCs, will deepen our understanding of the molecular basis of the efficient photosynthetic functions. The results obtained from these studies will be useful to develop novel artificial pigment-protein complexes. Developments of modified and *de novo* designed LHCs based on the structural and photofunctional mechanisms of native LHCs will lead to utilization of sunlight energy to tackle global problems on agricultural productivity and bioenergy production by improvement and/or redesign of photosynthesis (Ort et al. 2015, Croce et al. 2024).

## Supplementary Material

pcp-2025-e-00089-File009_pcaf084

## Data Availability

No new datasets were generated or analyzed in this study.
