# Supplementary material for: Chlorophyll reconstitution of photosynthetic light-harvesting complexes
Source: Plant Cell Physiol. 2025 Jul 28;66(11):1575–87. doi: 10.1093/pcp/pcaf084 (PMC12661315; doi:10.1093/pcp/pcaf084)
Supplement: pcp-2025-e-00089-File009_pcaf084 [file pcp-2025-e-00089-file009_pcaf084.pdf]

Supplementary materials

Chlorophyll Reconstitution of Photosynthetic Light-Harvesting Complexes

Yoshitaka Saga*, Shota Kawato, and Jiro Harada

Department of Chemistry, Faculty of Science and Engineering, Kindai University, Higashi-Osaka, Osaka 577-8502, Japan

Department of Medical Biochemistry, Kurume University School of Medicine, Fukuoka 830-0011, Japan

*Corresponding author: E-mail, saga@chem.kindai.ac.jp

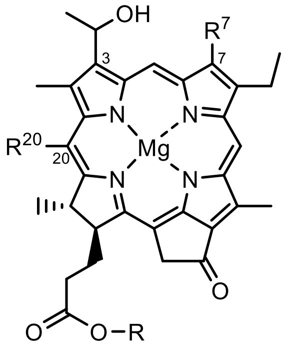

Pigment	R7	R20
BChl *c*	CH_3	CH_3
BChl *d*	CH_3	H
BChl *e*	CHO	CH_3
BChl *f*	CHO	H

Fig. S1. Molecular structures of chlorosomal BChl pigments in green photosynthetic bacteria.

Native polypeptide — Transmembrane α-helix

sphaeroides β ADKSDLGYTGL | TDEQAQELHSVYMSGLWLFSAVAIVAHLAVYIWR | PWF

Chemically synthesized polypeptide

sphaeroides β41 YTGLTDEQAQELHSVYMSGLWLFSAVAIVAHLAVYIWRPWF

sphaeroides β37 TDEQAQELHSVYMSGLWLFSAVAIVAHLAVYIWRPWF

sphaeroides β31 ELHSVYMSGLWLFSAVAIVAHLAVYIWRPWF

sphaeroides β16 VAIVAHLAVYIWRPWF

Fig. S2. Amino acid sequences of native LH1 β-polypeptide of *Rhodobacter sphaeroides* and examples of chemically synthesized polypeptides as analogs of the LH1 β-polypeptide used in the reconstitution studies of LH1. The sequences are aligned relative to the histidine residues (red) that are coordinated to BChl *a*.

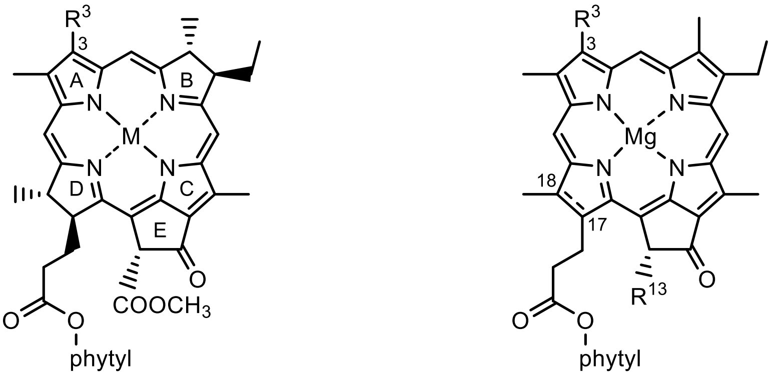

Pigment	R3	M
3-vinyl BChl *a*	$CHCH_2$	Mg
3^1-OH BChl *a*	$CH(OH)CH_3$	Mg
Zn BChl *a*	$COCH_3$	Zn

Pigment	R3	R13	C17-C18
3-acetyl Chl *a*	$COCH_3$	$COOCH_3$	single bond
pyro Chl *a*	$CHCH_2$	H	single bond
3-acetyl protoChl *a*	$COCH_3$	$COOCH_3$	double bond

phytyl = 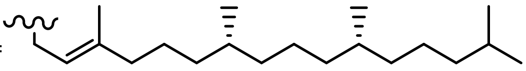

Fig. S3. Molecular structures of semi-synthetic (B)Chl pigments used in the reconstitution studies of LH2.

acidophilus LH2	-----------ATLTAEQSEELHKYVIDGTRVFLGLALVAHFLAFSATPWLH
acidophilus LH3	----------AEVLTSEQAEELHKHVIDGTRVFLVIAAIAHFLAFTLTPWLH
sphaeroides LH2	MTDDLNKVWPSGLTVAEAEEVHKQLILGTRVFGGMALIAHFLAAAATPWLG

Fig. S4. Amino acid sequences of β-polypeptides of LH2 and LH3 from purple photosynthetic bacteria *Rhodoblastus acidophilus* and *Rhodobacter sphaeroides*. The sequences are aligned relative to the histidine residues (red) that are coordinated to B850 BChl *a*. The arginine residues at position −10 with respect to the B850 ligand histidine are colored blue.
